# Supplementary material for: Structural and dynamic mechanisms of GABAA receptor modulators with opposing activities
Source: Nat Commun. 2022 Aug 6;13:4582. doi: 10.1038/s41467-022-32212-4 (PMC9357065; doi:10.1038/s41467-022-32212-4)
Supplement: Supplementary file 1 — Supplementary Information [file 41467_2022_32212_MOESM1_ESM.pdf]

Supplementary Information

Structural and dynamic mechanisms of $GABA_A$ receptor modulators with opposing activities

Shaotong Zhu[1,‡], Akshay Sridhar[2], Jinfeng Teng[1], Rebecca J. Howard[3], Erik Lindahl[2,3] and Ryan E. Hibbs[1, #]

[1]Departments of Neuroscience and Biophysics, University of Texas Southwestern Medical Center, Dallas, TX 75390, USA

[2]Dept. of Applied Physics, Science for Life Laboratory, KTH Royal Institute of Technology, Solna, Sweden

[3]Dept. of Biochemistry and Biophysics, Science for Life Laboratory, Stockholm University, Solna, Sweden

[‡]Current address: Institute for Protein Innovation, 4 Blackfan Circle, Boston, MA 02115, USA.

[#]Correspondence: ryan.hibbs@utsouthwestern.edu

This PDF file includes:

Supplementary Figures 1-6

Supplementary Table 1

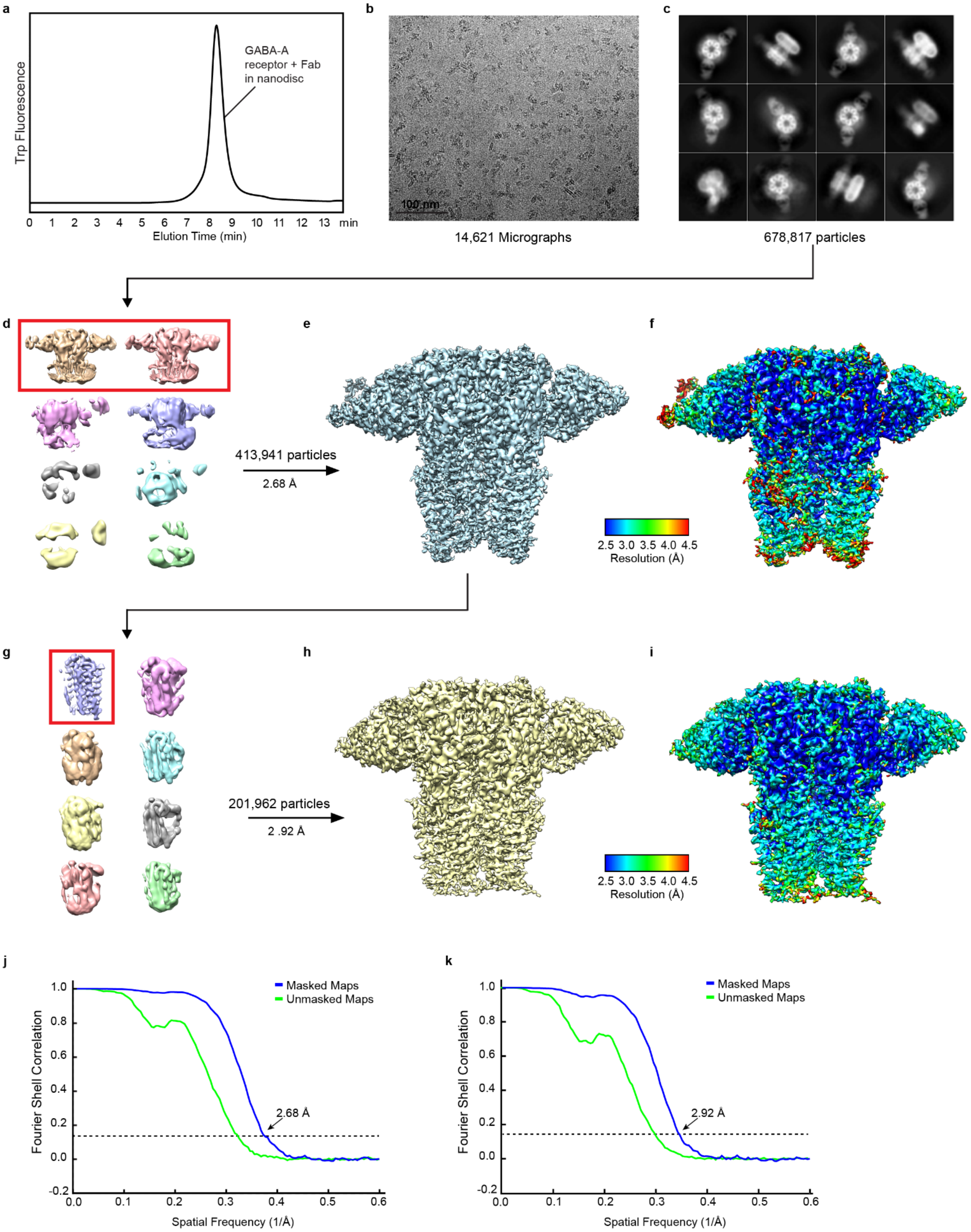
a
Trp Fluorescence
GABA-A
receptor + Fab
in nanodisc
0 1 2 3 4 5 6 7 8 9 10 11 12 13 min
Elution Time (min)
b
100 nm
14,621 Micrographs
c
678,817 particles
d
e
f
413,941 particles
2.68 Å
2.5 3.0 3.5 4.0 4.5
Resolution (Å)
g
h
i
201,962 particles
2 .92 Å
2.5 3.0 3.5 4.0 4.5
Resolution (Å)
j
Fourier Shell Correlation
Masked Maps
Unmasked Maps
2.68 Å
Spatial Frequency (1/Å)
k
Fourier Shell Correlation
Masked Maps
Unmasked Maps
2.92 Å
Spatial Frequency (1/Å)

Supplementary Figure 1. Cryo-EM image processing procedure for zolpidem complex.
a-i, Overview of the image processing procedure (see Methods). **a**, FSEC of $GABA_A$ receptor with Fab bound in nanodisc. **b,** Representative cryo-electron micrograph of the $GABA_A$ receptor-Fab complex. n=14,621 images. **c**, Images of selected 2D classes from reference-free 2D classification by Relion. **d**, 3D maps from a three-dimensional classification, from which particles from two classes (red boxes) were selected and used to generate map shown in **e**. **e,** Map after initial EM data processing with resolution 2.68 Å (at FSC = 0.143). **f,** Local resolution analysis of Map in **e** using ResMap. **g**, 3D maps from γ2-TMD focused classification, from which particles from one class (red box) were selected and used to generate the second map. **h,** The second map with better TMD density at overall resolution 2.92 Å. **i,** Local resolution analysis of the second map by ResMap. **j, k,** Fourier shell correlation (FSC) of two maps before (green) and after (blue) masking. **j,** Map in **e**.; **k,** Map in **h**.

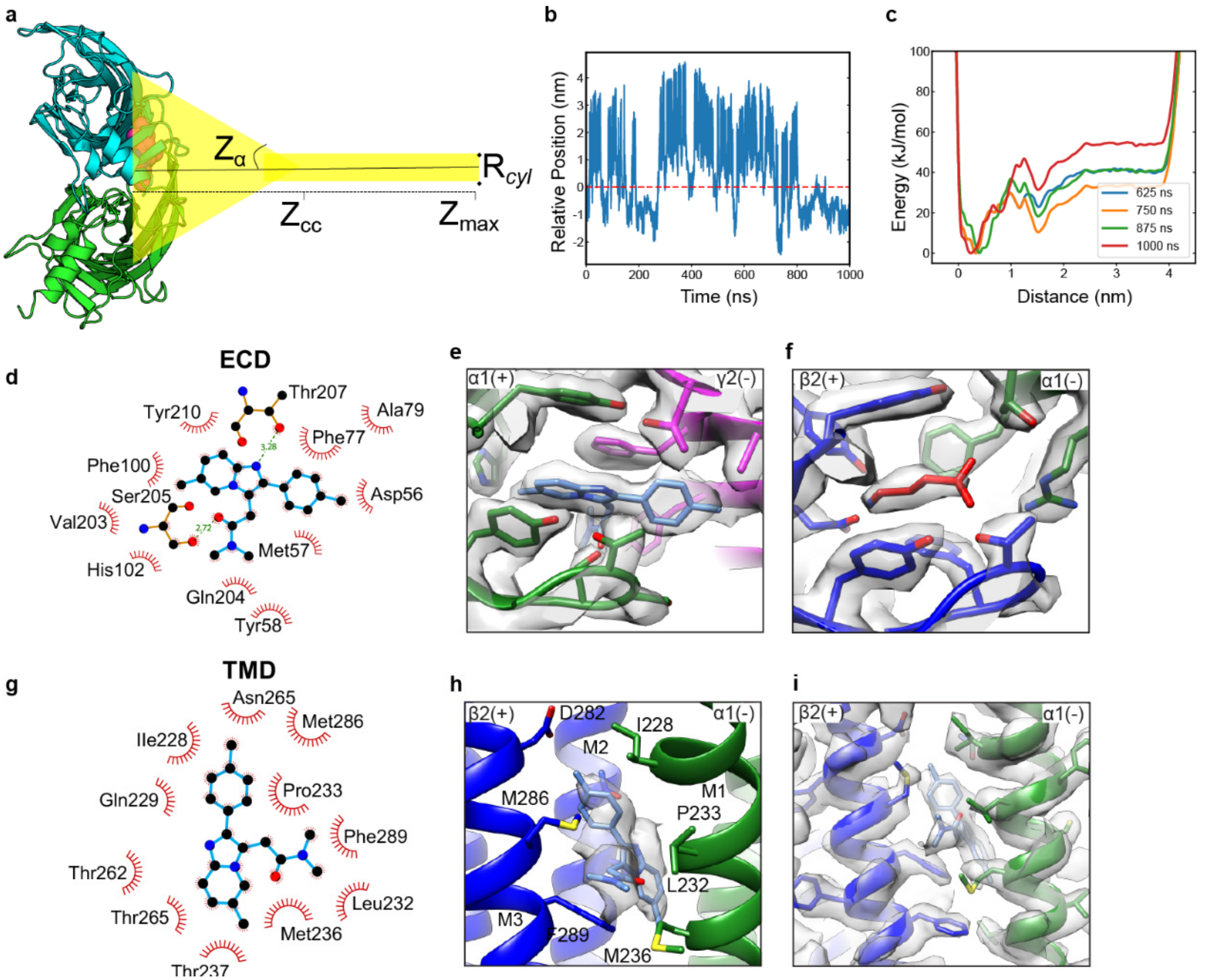

Supplementary Figure 2. Zolpidem interactions and representative map quality.
a-c, Funnel metadynamic calculation setup. **a,** Layout of the funnel metadynamics setup to investigate the ECD binding of ligands. The α1/γ2 subunits are colored green/cyan respectively and the ligand is shown in red. The funnel parameters Zα, Zcc, Zmax and Rcycl are set at 20°, 2.5 nm, 4.0 nm and 0.1 nm respectively. **b,** Assessing the convergence of the simulations through the number of binding and unbinding events. The relative position is calculated as a difference between the positions along the funnel vector of the ligand and the α1 loop C. **c,** Free-energy profile as function of simulation time along the biased distance collective variable. The profiles are normalized by setting the minimum to zero and are invariant disregarding the time-dependent constant. **d,** LigPlot schematic of zolpidem interactions in ECD showing electrostatic (dashes) and hydrophobic interactions (eyelashes). **e,** Map quality of zolpidem site at α/γ interface in ECD. **f**, Representative of map quality of GABA binding site at β/α interface. **g,** LigPlot schematic of zolpidem interactions in TMD. **h,** Second zolpidem binding site in the TMD at β/α interface. **i,** Map quality of zolpidem TMD binding site. Maps are shown as a transparent surface. Source data are provided as a Source Data file.

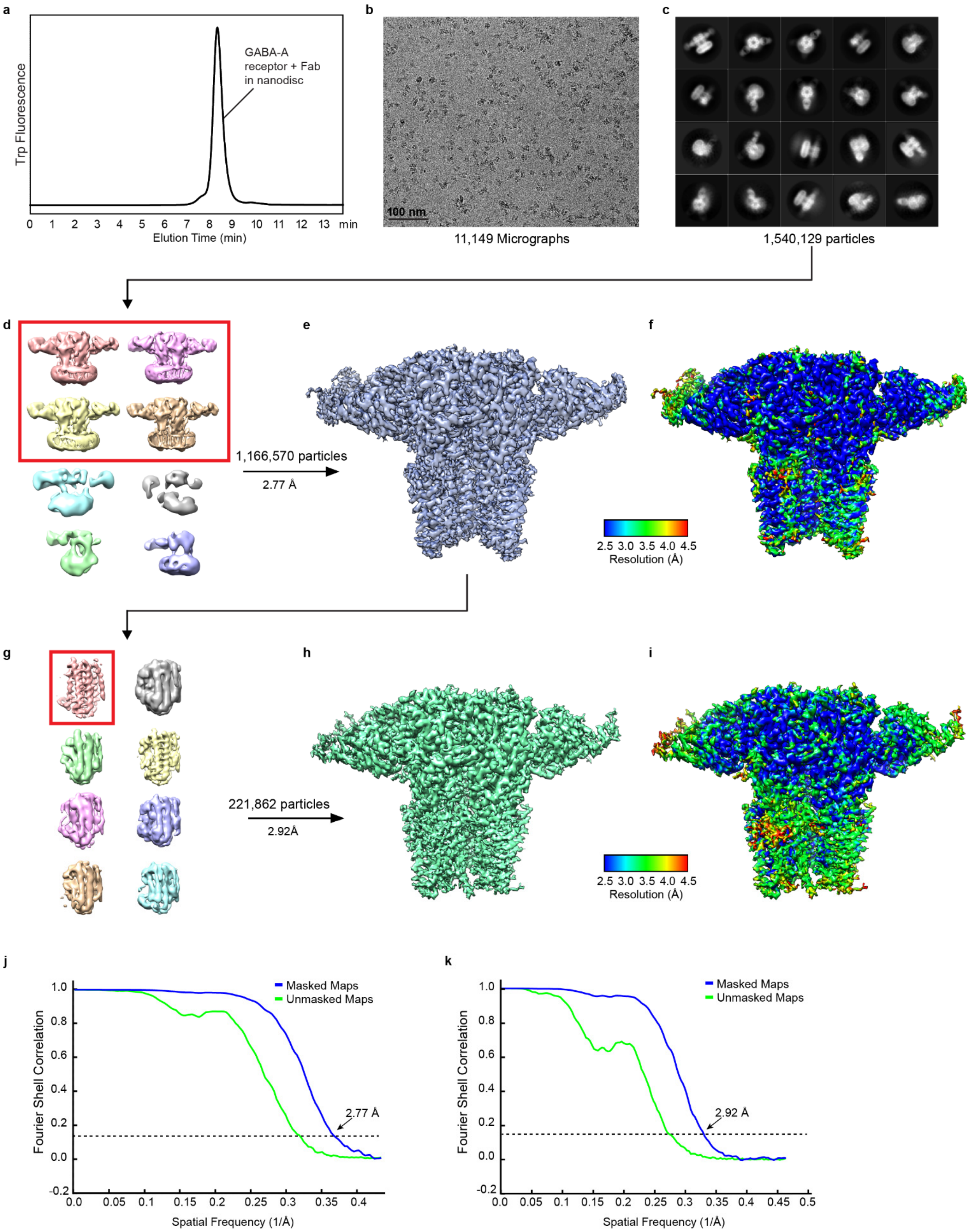
a
Trp Fluorescence
GABA-A receptor + Fab in nanodisc
0 1 2 3 4 5 6 7 8 9 10 11 12 13 min
Elution Time (min)
b
100 nm
11,149 Micrographs
c
1,540,129 particles
d
1,166,570 particles
2.77 Å
e
f
2.5 3.0 3.5 4.0 4.5
Resolution (Å)
g
221,862 particles
2.92Å
h
i
2.5 3.0 3.5 4.0 4.5
Resolution (Å)
j
Fourier Shell Correlation
Masked Maps
Unmasked Maps
2.77 Å
Spatial Frequency (1/Å)
k
Fourier Shell Correlation
Masked Maps
Unmasked Maps
2.92 Å
Spatial Frequency (1/Å)

Supplementary Figure 3. Cryo-EM image processing for DMCM complex.
a-i, Overview of the image processing procedure (see Methods). **a**, FSEC of $GABA_A$ receptor with Fab bound in nanodisc. **b,** Representative cryo-electron micrograph of the $GABA_A$ receptor-Fab complex. n=11,149 images. **c**, Images of selected two-dimensional classes from reference-free two-dimensional classification by Relion. **d**, Three-dimensional maps from a three-dimensional classification, from which particle from two classes (red boxes) were selected and used to generate map shown in **e**. **e,** Final EM map with resolution 2.77 Å. **f,** Local resolution analysis of the EM map as color code indicated in the bar by ResMap. **g**, 3D maps from a γ-subunit focused classification, from which particles from one class (red boxes) were selected and used to generate the second map. **h,** Second EM map with better TMD density at resolution 2.92 Å. **i,** Local resolution analysis of the second map by ResMap. **j, k,** Fourier shell correlation (FSC) of two maps before (green) and after (blue) masking. **j,** Map in **e**. **k,** Map in **h**.

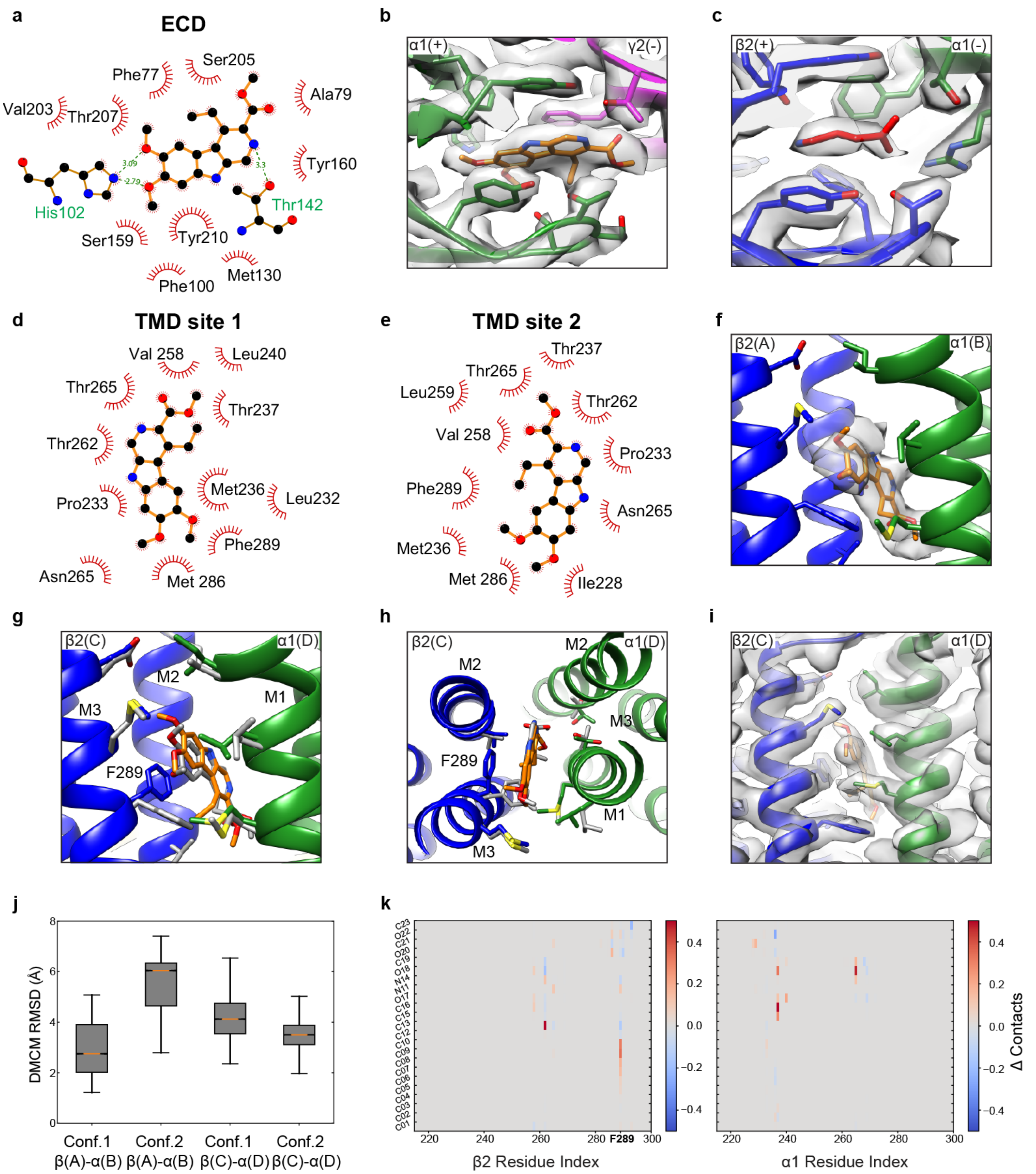

a
ECD
Phe77
Ser205
Ala79
Val203
Thr207
Tyr160
His102
Thr142
Ser159
Tyr210
Phe100
Met130
b
α1(+)
γ2(-)
c
β2(+)
α1(-)
d
TMD site 1
Val 258
Leu240
Thr265
Thr237
Thr262
Met236
Leu232
Pro233
Phe289
Asn265
Met 286
e
TMD site 2
Thr237
Thr265
Leu259
Thr262
Val 258
Pro233
Phe289
Asn265
Met236
Met 286
Ile228
f
β2(A)
α1(B)
g
β2(C)
α1(D)
M2
M1
M3
F289
h
β2(C)
M2
α1(D)
M3
F289
M1
M3
i
β2(C)
α1(D)
j
DMCM RMSD (Å)
Conf.1 β(A)-α(B)
Conf.2 β(A)-α(B)
Conf.1 β(C)-α(D)
Conf.2 β(C)-α(D)
k
β2 Residue Index
F289
α1 Residue Index
Δ Contacts

Supplementary Figure 4. DMCM interactions and representative map quality.
a, LigPlot schematic of DMCM interactions in ECD showing electrostatic (dashes) and hydrophobic interactions (eyelashes). **b, c,** Representatives of EM density map segments as transparent surfaces. **b,** DMCM site at α/γ interface in ECD. **c,** GABA binding site at β/α interface. **d, e,** LigPlot schematic of DMCM interactions in TMD. **d,** Site 1, at β(C)/α(D) interface. **e,** Site 2, at β(A)/α(B) interface. **f,** Second DMCM binding site in the TMD. **g, h,** Superposition of two DMCM binding sites in the TMD. Sidechain of β2-F289 is labeled. **g,** Side view. **h,** Top view. **i,** Map quality of DMCM site 1 at β(C)/α(D) interface. **j, k,** simulations of DMCM stability at two TMD binding sites. **j,** Stability of the two DMCM binding configurations at the two β2/α1 TMD subunit interfaces analyzed by the distribution of their rmsd. Data calculated from 10,000 frames and 3 simulations (n = 30,000). Boxes span the interquartile range (25th to 75th percentile) and whiskers extend up to 1.5 times. **k,** Comparison of the atomistic contacts guiding DMCM binding at the two β2/α1 TMD subunit interfaces. A contact was assumed if a non-hydrogen atom of the residue was within 3.2 Å of a ligand atom. A change in contact (Δ Contacts) was then calculated as the difference in contacts between the two interfaces. Source data are provided as a Source Data file.

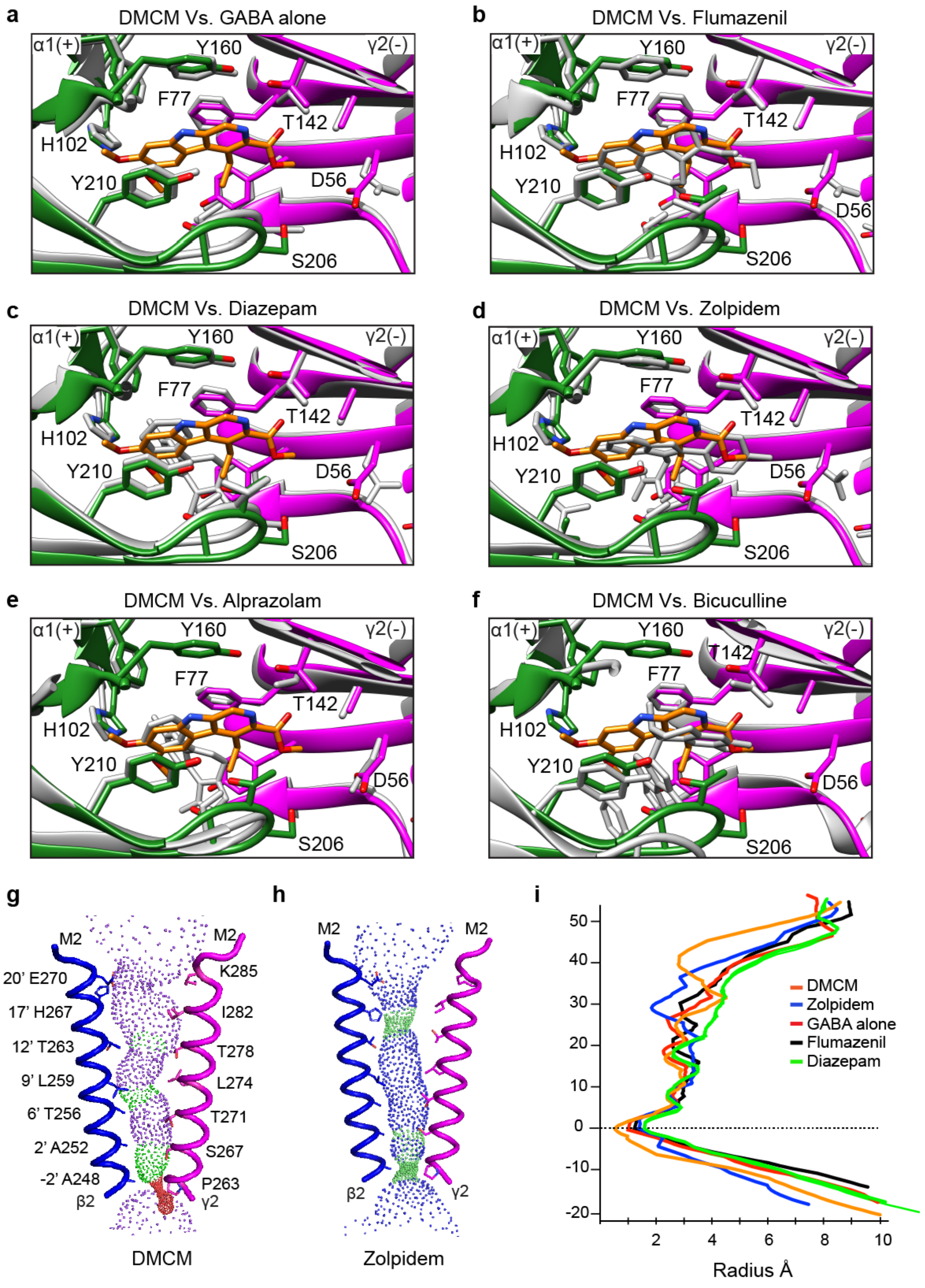
a
DMCM Vs. GABA alone
α1(+)
γ2(-)
Y160
F77
T142
H102
Y210
D56
S206
b
DMCM Vs. Flumazenil
α1(+)
γ2(-)
Y160
F77
T142
H102
Y210
D56
S206
c
DMCM Vs. Diazepam
α1(+)
γ2(-)
Y160
F77
T142
H102
Y210
D56
S206
d
DMCM Vs. Zolpidem
α1(+)
γ2(-)
Y160
F77
T142
H102
Y210
D56
S206
e
DMCM Vs. Alprazolam
α1(+)
γ2(-)
Y160
F77
T142
H102
Y210
D56
S206
f
DMCM Vs. Bicuculline
α1(+)
γ2(-)
Y160
T142
F77
H102
Y210
D56
S206
g
M2
M2
20' E270
17' H267
12' T263
9' L259
6' T256
2' A252
-2' A248
β2
K285
I282
T278
L274
T271
S267
P263
γ2
DMCM
h
M2
M2
β2
γ2
Zolpidem
i
50
40
30
20
10
0
-10
-20
2
4
6
8
10
Radius Å
DMCM
Zolpidem
GABA alone
Flumazenil
Diazepam

Supplementary Figure 5. Comparison of benzodiazepine site modulator binding modes and ion pore conformation. a, Superposition of empty benzodiazepine site (in gray, with only GABA bound) on DMCM bound structure. b, Superposition of flumazenil (in gray) on DMCM bound structure. c, Superposition of diazepam (in gray) on DMCM bound structure. d, Superposition of zolpidem (in gray) on DMCM bound structure. e, Superposition of alprazolam (in gray) (PDB: 6HUO [http://dx.doi.org/10.2210/pdb6huo/pdb]) on DMCM bound structure. f, Superposition of bicuculline (in gray) on DMCM bound structure. g, h, Pore conformations for α1β2γ2 GABAA receptors structures bound by g, DMCM plus GABA. h, zolpidem plus GABA. i, Analysis of pore diameter; y-axis is distance along pore axis.

a

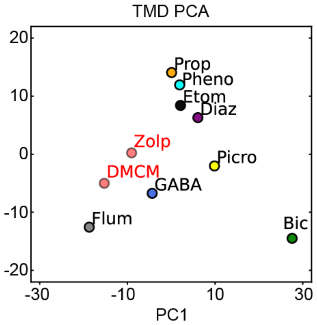

b

RMSD (Å)	GABA	Diaz	Flum	DMCM or Zolp	Picro	Bic	Prop	Pheno	Etom
Zolpidem & GABA	0.54	0.42	0.97	0.58	0.61	1.03	0.45	0.50	0.44
DMCM & GABA	0.79	0.51	0.72	0.58	0.79	0.97	0.46	0.75	0.51

c

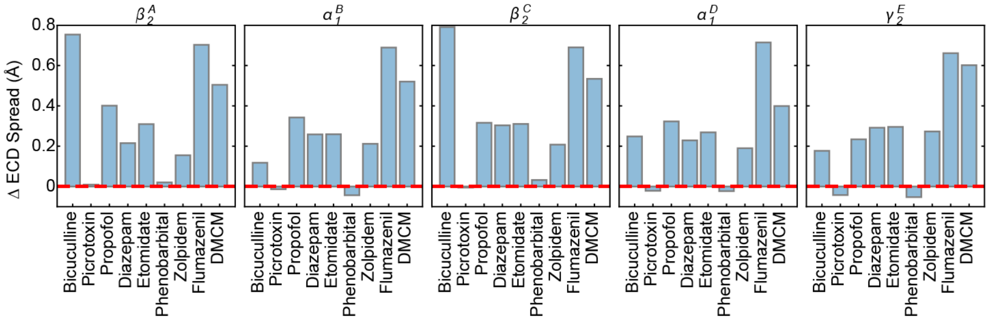

d

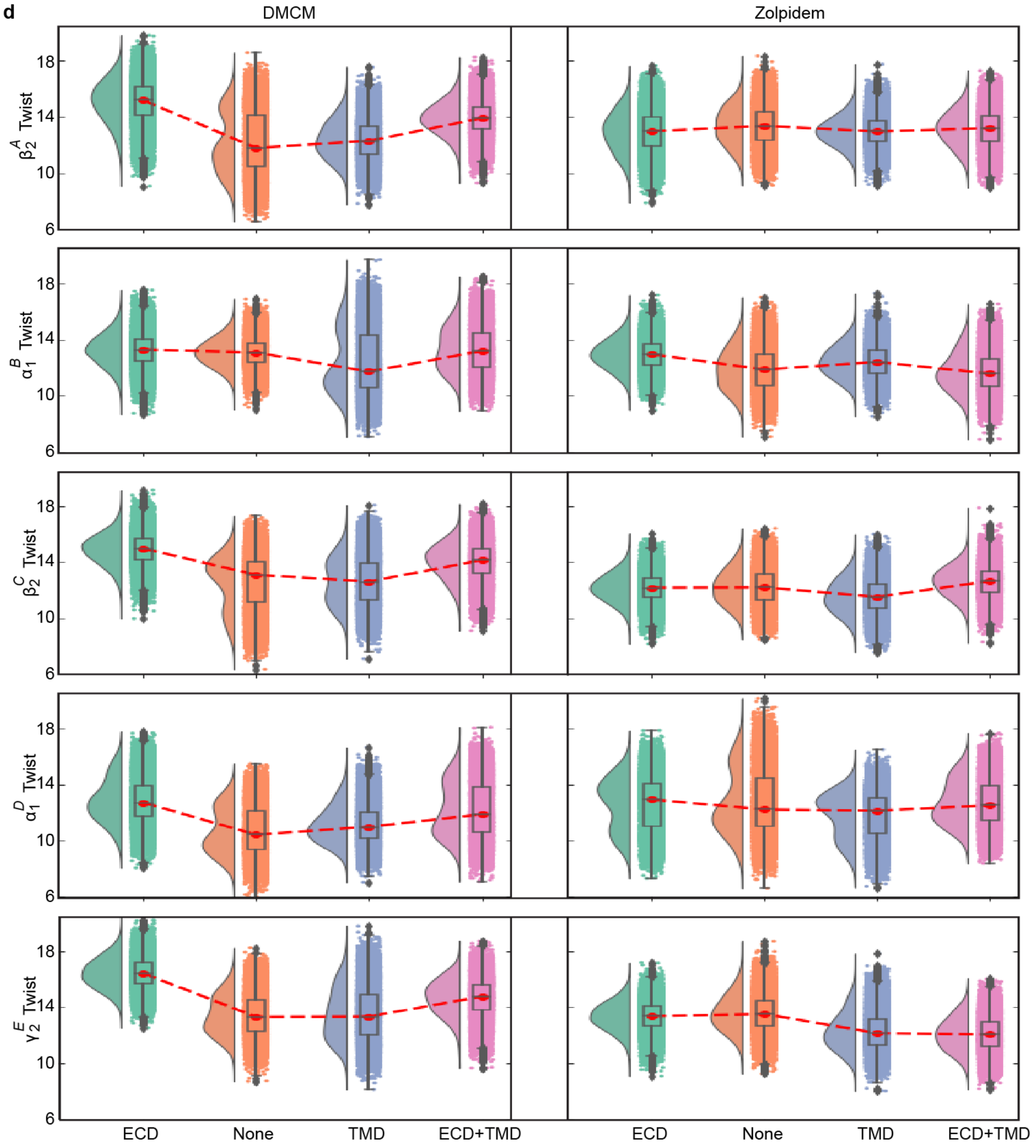

Supplementary Figure 6. MD simulations of benzodiazepine-site ligand modulation.
a, Dominant principal components of the zolpidem and DMCM structures analyzed with the 8 previously resolved α1β2γ2 structures calculated within the TMD. **b,** RMSD analysis of the new Zolpidem and DMCM structures with 8 previously resolved α1β2γ2 structures. **c,** The ECD spread of five subunits within structures resolved with bound PAMs/NAMs calculated as the distance between the subunit's Cα atoms center-of-mass and that of the entire structure's ECD. The spread values are normalized relative to the spread within the structure resolved with only GABA bound (PDB: 6X3Z [http://dx.doi.org/10.2210/pdb6x3z/pdb]). **d,** Raincloud plots quantifying the relative orientations of the extracellular and transmembrane domains of the five subunits from MD simulations varying ligand binding. The domain twist is a rotational angle measured as in Fig. 5 and described in the Methods. Probability distributions are plotted on the left and raw data (n = 24,000; 8,000 frames each from 3 simulations) within boxplots on the right. Boxes span the interquartile range (25th to 75th percentile), whiskers extend up to 1.5 times and outliers are plotted as dots. The median values from each simulation condition are joined by red dotted lines to illustrate differences between them. Source data are provided as a Source Data file.

Supplementary Table 1. Cryo-EM data collection, refinement and validation statistics

	**GABA & Zolpidem**<br>EMDB-27332<br>PDB 8DD2	**GABA & DMCM**<br>EMDB-27333<br>PDB 8DD3
**Data collection**		
Magnification	105K	81K
Voltage (ke⁻V)	300	300
Electron exposure (e⁻/Å^2)	55.7	50
Defocus range (μm)	-0.8 to -2.2	-0.8 to -2.2
Pixel size (Å/pixel)	0.834	1.079
Symmetry imposed	C1	C1
Initial particle images (no.)	2,411,674	2,092,528
Final particle images (no.)	201,962	221,862
Map Resolution (Å)	2.92	2.92
FSC threshold	0.143	0.143
Map Resolution range (Å)	2.5 to 3.5	2.5 to 3.5
**Refinement**		
Initial model used (PDB code)	6X3X	6X3X
Model Resolution (Å)	2.9	3.0
FSC threshold	0.5	0.5
Model Resolution range (Å)	n.a.	n.a.
Map-sharpening B factor (Å^2)	-50	-50
Model composition		
Number of non-H atoms	17,209	17,211
Protein residues	2,121	2,121
Ligand	28	28
B factor (Å^2)		
Protein	52.75	67.26
Ligand	49.44	63.79
R.m.s.d. values		
Bond lengths (Å)	0.004	0.005
Bond angles (Å)	0.54	0.56
Validation		
Molprobity score	1.62(100thpercentile)	1.58 (100thpercentile)
Clashscore	5.62 (100thpercentile)	6.06 (100thpercentile)
Poor rotamers (%)	0.0	0.0
Ramachandran analysis		
Favored (%)	95.48	96.29
Allowed (%)	4.52	3.71
Outliers (%)	0	0
