# Supplementary material for: Structural and dynamic mechanisms of GABAA receptor modulators with opposing activities
Source: Nat Commun. 2022 Aug 6;13:4582. doi: 10.1038/s41467-022-32212-4 (PMC9357065; doi:10.1038/s41467-022-32212-4)
Supplement: Supplementary file 3 — Reporting Summary [file 41467_2022_32212_MOESM3_ESM.pdf]

nature portfolio

Corresponding author(s): Ryan Hibbs

Last updated by author(s): 7/5/2022

Reporting Summary

Nature Portfolio wishes to improve the reproducibility of the work that we publish. This form provides structure for consistency and transparency in reporting. For further information on Nature Portfolio policies, see our Editorial Policies and the Editorial Policy Checklist.

Statistics

For all statistical analyses, confirm that the following items are present in the figure legend, table legend, main text, or Methods section.

n/a	Confirmed	
☐	☒	The exact sample size (*n*) for each experimental group/condition, given as a discrete number and unit of measurement
☐	☒	A statement on whether measurements were taken from distinct samples or whether the same sample was measured repeatedly
☐	☒	The statistical test(s) used AND whether they are one- or two-sided<br>*Only common tests should be described solely by name; describe more complex techniques in the Methods section.*
☒	☐	A description of all covariates tested
☒	☐	A description of any assumptions or corrections, such as tests of normality and adjustment for multiple comparisons
☐	☒	A full description of the statistical parameters including central tendency (e.g. means) or other basic estimates (e.g. regression coefficient) AND variation (e.g. standard deviation) or associated estimates of uncertainty (e.g. confidence intervals)
☐	☒	For null hypothesis testing, the test statistic (e.g. *F*, *t*, *r*) with confidence intervals, effect sizes, degrees of freedom and *P* value noted<br>*Give P values as exact values whenever suitable.*
☒	☐	For Bayesian analysis, information on the choice of priors and Markov chain Monte Carlo settings
☒	☐	For hierarchical and complex designs, identification of the appropriate level for tests and full reporting of outcomes
☒	☐	Estimates of effect sizes (e.g. Cohen's *d*, Pearson's *r*), indicating how they were calculated

Our web collection on statistics for biologists contains articles on many of the points above.

Software and code

Policy information about availability of computer code

Data collection	SerialEM 3.8, pCLAMP 10.7
Data analysis	Relion 3.1, Phenix 1.17.1-3660, UCSF Chimera 1.15, Pymol 2.5.1, GraphPad Prism 9.1.2, Molprobity 4.5.1, Coot 0.8.9.2, Hole2, SBGrid 2.2.11, LigPlot+ v.1.4.5, MotionCor2, GCTF v1.06, CTFFIND4.1, crYOLO 1.6.0, ResMap, GROMACS 2019.6, CHARMM-GUI, VMD 1.9.4, MDAnalysis 2.0.0, Plumed 2.7.0

For manuscripts utilizing custom algorithms or software that are central to the research but not yet described in published literature, software must be made available to editors and reviewers. We strongly encourage code deposition in a community repository (e.g. GitHub). See the Nature Portfolio guidelines for submitting code & software for further information.

Data

Policy information about availability of data

All manuscripts must include a data availability statement. This statement should provide the following information, where applicable:

- Accession codes, unique identifiers, or web links for publicly available datasets
- A description of any restrictions on data availability
- For clinical datasets or third party data, please ensure that the statement adheres to our policy

Data availability: Atomic model coordinates for GABA + zolpidem and GABA + DMCM–bound structures have been deposited in the Protein Data Bank with accession codes 8DD2 and 8DD3, respectively, and the cryo-EM density maps have been deposited in the Electron Microscopy Data Bank with accession codes EMD-27332 and EMD-27333, respectively.

Field-specific reporting

Please select the one below that is the best fit for your research. If you are not sure, read the appropriate sections before making your selection.

☒ Life sciences ☐ Behavioural & social sciences ☐ Ecological, evolutionary & environmental sciences

For a reference copy of the document with all sections, see nature.com/documents/nr-reporting-summary-flat.pdf

Life sciences study design

All studies must disclose on these points even when the disclosure is negative.

Sample size	No statistical methods were used to estimate appropriate sample size. Size of cryo-EM dataset sample size was based on sufficient number of images and particles to obtain a high resolution reconstruction.
Data exclusions	No data were excluded from the analyses.
Replication	All electrophysiological data were replicated at least 3 times using independent cells and were performed over different days of recording. Attempts to replicate/reproduce the data were successful as detailed in the MD simulation and electrophysiology replicates.
Randomization	EM particle sets were randomly split for the purposes of estimating overall resolution.
Blinding	None.

Reporting for specific materials, systems and methods

We require information from authors about some types of materials, experimental systems and methods used in many studies. Here, indicate whether each material, system or method listed is relevant to your study. If you are not sure if a list item applies to your research, read the appropriate section before selecting a response.

Materials & experimental systems

n/a	Involved in the study	
☐	☒	Antibodies
☐	☒	Eukaryotic cell lines
☒	☐	Palaeontology and archaeology
☒	☐	Animals and other organisms
☒	☐	Human research participants
☒	☐	Clinical data
☒	☐	Dual use research of concern

Methods

n/a	Involved in the study	
☒	☐	ChIP-seq
☒	☐	Flow cytometry
☒	☐	MRI-based neuroimaging

Antibodies

Antibodies used	The 1F4 monoclonal antibody was raised using standard methods at Monoclonal Core, Vaccine and Gene Therapy Institute, Oregon Health & Science University, described in pubmed ID: 29950725.
Validation	Binding was validated by gel filtration and structural analysis; sequence was verified by sequencing of hybridoma line and by experimental density map.

Eukaryotic cell lines

Policy information about cell lines

Cell line source(s)	HEK293S GnTI- was purchased from ATCC (CRL-3022).
Authentication	Purchased from commercial supplier. Not further tested.
Mycoplasma contamination	Tested negative by ATCC.
Commonly misidentified lines (See ICLAC register)	No commonly misidentified cell lines were used.
